# Through Thick and Thin: Baseline Cortical Volume and Thickness Predict Performance and Response to Transcranial Direct Current Stimulation in Primary Progressive Aphasia

**DOI:** 10.3389/fnhum.2022.907425

**Published:** 2022-07-07

**Authors:** Nicole R. Nissim, Denise Y. Harvey, Christopher Haslam, Leah Friedman, Pandurang Bharne, Geneva Litz, Jeffrey S. Phillips, Katheryn A. Q. Cousins, Sharon X. Xie, Murray Grossman, Roy H. Hamilton

**Affiliations:** ^1^Laboratory for Cognition and Neural Stimulation, Department of Neurology, University of Pennsylvania, Philadelphia, PA, United States; ^2^Moss Rehabilitation Research Institute, Elkins Park, PA, United States; ^3^Department of Neurology, University of Pennsylvania, Philadelphia, PA, United States; ^4^Penn Frontotemporal Degeneration Center, University of Pennsylvania, Philadelphia, PA, United States; ^5^Department of Biostatistics, Epidemiology and Informatics, University of Pennsylvania, Philadelphia, PA, United States

**Keywords:** transcranial direct current stimulation (tDCS), primary progressive aphasia (PPA), constraint induced language therapy (CILT), structural neuroimaging, western aphasia battery-revised (WAB-R)

## Abstract

**Objectives:**

We hypothesized that measures of cortical thickness and volume in language areas would correlate with response to treatment with high-definition transcranial direct current stimulation (HD-tDCS) in persons with primary progressive aphasia (PPA).

**Materials and Methods:**

In a blinded, within-group crossover study, PPA patients (*N* = 12) underwent a 2-week intervention HD-tDCS paired with constraint-induced language therapy (CILT). Multi-level linear regression (backward-fitted models) were performed to assess cortical measures as predictors of tDCS-induced naming improvements, measured by the Western Aphasia Battery-naming subtest, from baseline to immediately after and 6 weeks post-intervention.

**Results:**

Greater baseline thickness of the pars opercularis significantly predicted naming gains (*p* = 0.03) immediately following intervention, while greater thickness of the middle temporal gyrus (MTG) and lower thickness of the superior temporal gyrus (STG) significantly predicted 6-week naming gains (*p*’s < 0.02). Thickness did not predict naming gains in sham. Volume did not predict immediate gains for active stimulation. Greater volume of the pars triangularis and MTG, but lower STG volume significantly predicted 6-week naming gains in active stimulation. Greater pars orbitalis and MTG volume, and lower STG volume predicted immediate naming gains in sham (*p*’s < 0.05). Volume did not predict 6-week naming gains in sham.

**Conclusion:**

Cortical thickness and volume were predictive of tDCS-induced naming improvement in PPA patients. The finding that frontal thickness predicted immediate active tDCS-induced naming gains while temporal areas predicted naming changes at 6-week suggests that a broader network of regions may be important for long-term maintenance of treatment gains. The finding that volume predicted immediate naming performance in the sham condition may reflect the benefits of behavioral speech language therapy and neural correlates of its short-lived treatment gains. Collectively, thickness and volume were predictive of treatment gains in the active condition but not sham, suggesting that pairing HD-tDCS with CILT may be important for maintaining treatment effects.

## Introduction

Primary progressive aphasia (PPA) is a debilitating syndrome marked by progressive loss of language skills resulting from underlying neurodegenerative diseases. Unfortunately, speech language therapy (SLT), the current standard-of-care, is only modestly beneficial (Beeson et al., 2011; Tippett et al., 2015; Gervits et al., 2017). A growing body of evidence suggests that transcranial direct current stimulation (tDCS), a form of non-invasive neuromodulation, can enhance language outcomes in persons with PPA when paired with SLT (Tsapkini et al., 2014; Gervits et al., 2017; Fenner et al., 2019; Cotelli et al., 2020; Nissim et al., 2020). TDCS involves the delivery of weak electrical currents via electrodes placed on the scalp (Nitsche and Paulus, 2000), which alter the resting membrane potential of neurons and enable the modulation of neuronal excitability (facilitation or suppression) of targeted cortical regions (Nitsche and Paulus, 2000; Nitsche et al., 2005; Vecchio et al., 2016). TDCS has shown promise in facilitating behaviorally beneficial neuroplasticity when targeting a brain region responsible for a particular cognitive process, particularly when paired with a task that engages the targeted brain system (Gill et al., 2015; Pellicciari and Miniussi, 2018; Dubreuil-Vall et al., 2019; Kronberg et al., 2020). However, responses to tDCS vary considerably, which limits its advancement as a potential intervention for persons with PPA. The causes of this variability remain poorly understood (Datta et al., 2012; Horvath et al., 2014; Tsapkini et al., 2014), in part because characteristics that predict responsiveness to tDCS treatment in PPA patients are relatively unexplored.

A pathological hallmark of PPA is cortical atrophy in key regions of the brain’s language network (Mesulam, 2003; Sreepadma et al., 2003; Gorno-Tempini et al., 2011; Rogalski et al., 2011; Norise and Hamilton, 2017). Different patterns of cortical atrophy are observed in the three known subtypes of PPA. Atrophy is observed in the left inferior frontal lobe and insula in persons with non-fluent/agrammatic PPA (naPPA), who experience difficulties with language composition at the level of words and grammar. Individuals with logopenic variant PPA (lvPPA) show atrophy of the left posterior temporal and parietal lobes, and experience difficulties in lexical retrieval and phonological loop functions. Semantic variant PPA (svPPA) involves anterior and ventral temporal lobe atrophy that account for deficits in processing conceptual information (Grossman and Ash, 2004; Grossman, 2012, 2018; Agosta et al., 2013; Giannini et al., 2017).

A gap in knowledge exists in understanding the contribution of both cortical thickness and volume atrophy and its relationship to tDCS-related language gains in PPA. Here, we are interested in exploring whether cortical thickness and/or volume across PPA subtypes may be predictive of tDCS-induced language gains. We operationalize cortical volume and thickness, where the former is defined as the amount of gray matter that lies between the gray-white boundary and the pia mater and the latter as the width of gray matter structure (i.e., cortical ribbon), respectively (Winkler et al., 2010; Nissim et al., 2017). Volume is quantified by both thickness and surface area and represents of the amount and size of neurons and dendritic processes (Makris et al., 2007; Dickerson and Wolk, 2012). Thickness serves as a marker of the integrity of the cerebral cortex and relates to the size, number, and density of cells in a cortical column (Rakic, 1988). Numerous studies have shown that cortical thickness is an important parameter related with cognitive abilities (Engvig et al., 2010; McGugin et al., 2016; Schmidt et al., 2016), and thinner cortex is a reliable index of atrophy in neurodegenerative diseases (Dickerson et al., 2009). Yet, to our knowledge, prior research in PPA has largely focused on cortical volume as a predictor of response to tDCS-induced language gains (Cotelli et al., 2016; de Aguiar et al., 2020). We hypothesized that the degree of cortical thickness and volume atrophy in language-relevant brain regions could strongly influence tDCS treatment response in persons with PPA.

There are at least two reasons to suspect that cortical atrophy could be a strong predictor of tDCS efficacy in persons with PPA. First, because tDCS-induced long-term changes in brain function are thought to be mediated by modulation of synaptic plasticity (Monte-Silva et al., 2013; Rahman et al., 2013; Pelletier and Cicchetti, 2015), they are theoretically dependent on the density of synapses and overall neuronal connectivity in stimulated areas, both of which are markedly reduced in brain regions that have undergone neurodegenerative atrophy (Pini et al., 2016, 2018). Secondly, the amount and distribution of current that reaches brain tissue during tDCS administration may depend on individual anatomy (Kim et al., 2014; Unal et al., 2020). Some computational modeling studies have suggested that cortical atrophy (i.e., decreased gray matter and increased cerebrospinal fluid) reduces the current density that reaches atrophied parts of the brain (Mahdavi and Towhidkhah, 2018), although more recent studies have called this finding into question (Unal et al., 2020). Thus, cortical atrophy may reduce the effects of tDCS, both because atrophied areas contain less substrate to stimulate and likely receive less current than relatively spared brain regions.

The potential of tDCS to elicit adaptive neuroplasticity in patients with aphasia has previously been associated with two possible and not mutually exclusive mechanisms: (1) the activation (or re-activation) of canonical networks that become dysfunctional due to disease pathology; or (2) the recruitment of compensatory networks that engage brain regions previously uninvolved in language processing (Chrysikou and Hamilton, 2011; Cotelli et al., 2016). Current research in persons with PPA supports the notion that tDCS engages pre-existing neural substrates within the damaged language network, as evidence shows that partially spared cortical regions involved in language are essential to confer tDCS-induced language enhancement (Turkeltaub et al., 2011; Norise and Hamilton, 2017). This has been demonstrated in prior PPA studies focusing on naming improvement, where cortical volume predicted conventional tDCS-induced improvements after a written naming and spelling therapy (de Aguiar et al., 2020) and Individualized Computerized Anomia Training (ICAT; Cotelli et al., 2016).

In the current double-blind, within-subject crossover exploratory pilot study, we examined whether baseline cortical thickness (i.e., width of cortical gray matter) and/or volume (i.e., a representation of the amount and size of neurons and dendritic processes) of left hemisphere language regions predict improvements in language abilities after a 2-week intervention of active versus sham high-definition tDCS (HD-tDCS) in PPA patients. Cortical thinning corresponds well with clinical manifestations in PPA (Thanprasertsuk and Likitjaroen, 2021), while cortical volume atrophy has shown associations with lower naming accuracy in PPA patients (Meyer et al., 2017); thus, we were interested in examining the relationship between tDCS-induced naming improvement and cortical measures at baseline. HD-tDCS, which affords increased stimulation focality, and to our knowledge, has not yet been explored in PPA, was paired with constraint induced language therapy (CILT) during ten daily sessions. CILT was selected as it has previously demonstrated to be efficacious in persons with PPA (Hameister et al., 2017). Language abilities were assessed using the Western Aphasia Battery-Revised (WAB-R; Kertesz, 2006) at baseline, immediately after intervention (0-week) and 6-week post-intervention to assess maintenance of tDCS-induced treatment gains. We hypothesized that left hemisphere language regions with greater cortical thickness or volume at baseline would predict language gains at post-intervention time points in the active condition, but not sham, when compared to cortical atrophy of a control site and/or cortical atrophy more generally (i.e., global cortical volume/thickness).

## Materials and Methods

### Participants

Twelve individuals with PPA (lvPPA = 8, svPPA = 2, naPPA = 2) were included in this study [4 females; mean age = 66.92(±6.37); mean education = 17.17(±1.95)]. Participants were recruited from the Penn Frontotemporal Degeneration Center (FTDC) and were diagnosed according to Gorno-Tempini et al. (2011) guidelines. At the time of enrollment, mean time since diagnosis was 3.42(±1.71) years. Participants were enrolled if they met the following inclusion criteria: right-handedness, native English-speakers, no contraindications to undergoing tDCS or MRI, no other active neurologic conditions, and a Mini Mental Status Examination (MMSE; Folstein et al., 1975) score ≥15 prior to enrollment [mean MMSE = 24.5(±2.88); range = 19–29] to ensure that cognitive impairment did not impede engagement in the study protocol. All participants provided informed consent in accordance with the University of Pennsylvania’s Institutional Review Board (IRB). Table 1 summarizes demographic data.

**TABLE 1:** Demographics and characteristics of each participant and the total sample (mean age, sex, PPA subtype, years since onset, baseline MMSE score, order of stimulation condition).

ID	Sex	Age	Subtype	Disease onset (years)	MMSE at baseline	First stimulation condition
1	M	71	lvPPA	6	25	Active
2	M	58	lvPPA	2	26	Sham
3	M	77	naPPA	3	25	Active
4	F	55	lvPPA	2	19	Active
5	M	69	lvPPA	3	25	Sham
6	M	63	svPPA	7	23	Active
7	F	71	svPPA	3	24	Active
8	F	69	naPPA	4	23	Sham
9	F	68	lvPPA	3	25	Active
10	M	72	lvPPA	2	29	Sham
11	M	59	lvPPA	5	21	Active
12	M	71	lvPPA	1	29	Sham
Total	4F; 8M	66.92	8 lvPPA; 2 svPPA; 2 naPPA	3.42	24.5	7 active first; 5 sham first

### Intervention Timeline

**FIGURE 1:**
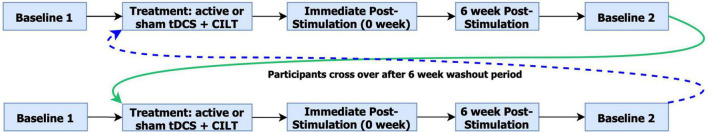
Overview of study design. The color differentiation indicates arm 1 crossover to arm 2 and arm 2 crossover to arm 1.

Participants were randomly assigned to receive a 10-day course of CILT for 1 h paired with 20 min of active (or sham) HD-tDCS in the first (or second) arm (counterbalanced for stimulation condition). Participants (*N* = 12) completed the WAB-R prior to each treatment arm and again immediately after (0-week) and 6-week post-intervention. To avoid carryover effects, arm 2 baseline assessment was administered 12-week following arm 1 treatment. One participant (ID#12) was excluded from behavioral analyses due to missing the 6-week time point, but was included in imaging analyses. Both treatment arms followed identical procedures, differing only with respect to the stimulation condition (see Figure 1). The FTDC obtained T1-weighted MRI scans and administered the MMSE within ∼7 months prior to enrollment. At enrollment, the MMSE was readministered. A paired-samples *t*-test revealed no significant decline in cognitive status from the time of the scan and enrollment in the study [*t*(11) = −1.43, *p* = 0.2].

### Neuroimaging Acquisition and Processing

Participants underwent structural neuroimaging on a 3-Tesla Siemens magnetic resonance imaging (MRI) scanner. MRI data for 6 participants were collected with a T1-weighted sagittal MP-RAGE sequence with in-plane voxel sizes of 1.05 mm × 1.05 mm, slice thickness = 1.2 mm, repetition time = 2300 ms, echo time = 2.91 ms, and inversion time = 900 ms. The remaining participants underwent a multi-echo T1-weighted MPRAGE with in-plane voxel sizes of 0.8 mm × 0.8 mm, slice thickness = 0.8 mm, repetition time = 2400 ms, inversion time = 1020 ms, and echo times of 1.96 ms, 3.88 ms, 5.8 ms, and 7.72 ms (Tisdall et al., 2016). This difference in scanner parameters is within an acceptable deviation range (Mulder et al., 2019), and therefore likely does not affect analyses conducted on the full sample. Images were processed using Advanced Normalization Tools (ANTs), which has been extensively validated against surface-based methods such as FreeSurfer (Tustison et al., 2014). Images underwent intensity normalization and were spatially normalized to a healthy control template from the Open Access Series of Imaging Studies (OASIS) dataset using a symmetric diffeomorphic algorithm (Klein et al., 2009; Avants et al., 2011; Phillips et al., 2018). Images were then segmented into six tissue classes using template-based priors: cortical gray matter, deep gray matter, white matter, CSF, brainstem, and cerebellum. Tissue segmentation posteriors were then used to estimate cortical thickness. The Lausanne atlas (scale 60) was aligned to each T1-weighted image and intersected with gray matter probability maps to obtain gray matter volume estimates for each parcellated region. Volume was computed from voxels in each region with a gray matter probability of 50% or higher. Gray matter volumes were normalized by intracranial volume and converted to z-scores based on gray matter volume in control images. All scans were visually inspected for image quality, and outlier analyses were performed (i.e., *z*-scores > 3.0) to identify extreme values due to segmentation error or other artifacts.

### Brain Regions of Interest (ROIs)

**FIGURE 2:**
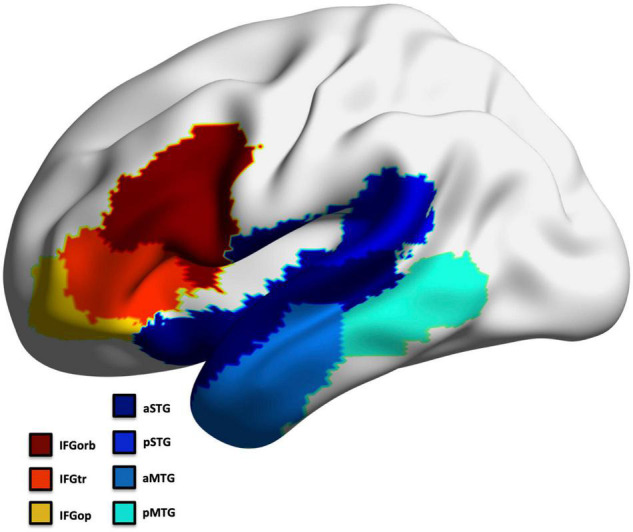
Left hemisphere language ROIs.

To avoid multiple comparison issues, we selected *a priori* seven regions that exhibit predominant pathology across all PPA subtypes in the frontal and temporal left hemisphere to represent the language network (Mesulam et al., 2014; Thanprasertsuk and Likitjaroen, 2021; Figure 2). This included regions involved in confrontation naming, speech production, comprehension, and general language processing (Ojemann et al., 1988; Kan and Thompson-Schill, 2004; Newhart et al., 2007; MacDonald et al., 2015; Walenski et al., 2019): inferior frontal gyrus (IFG) parcellated into the pars opercularis (IFGop), pars orbitalis (IFGorb), and pars triangularis (IFGtr), middle temporal gyrus (MTG) and superior temporal gyrus parcellated into an anterior and posterior region (aMTG; pMTG; aSTG; pSTG). The left occipital cortex was assessed as a control region (separately for thickness and volume; divided into 5 ROIs).

### High-Definition tDCS (HD-tDCS) Parameters

**FIGURE 3:**
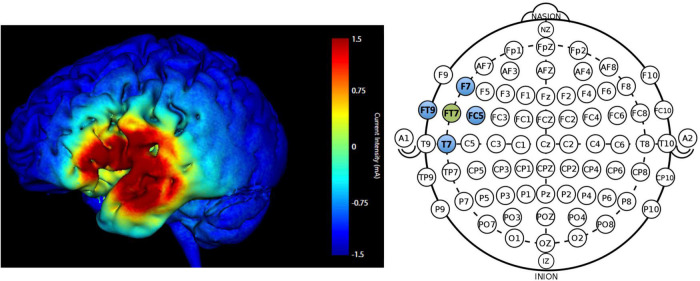
Predicted current modeling simulation at 1.5 mA intensity for electrode placement at FT7 (anode; green) and F7, T7, FC5, FT9 (cathode electrodes; blue). The color legend indicates current intensity (warmer colors = higher current intensity; max = 1.5 mA).

Participants received HD-tDCS (SoterixMedical 1 × 1 low intensity transcranial DC Stimulator and 4 × 1 Multi-Channel Stimulation Interface) at 1.5 mA intensity for 20-min in the active condition with 30 s current ramp-up and -down. Electrodes were placed following the International 10-10 EEG system. The montage targeted the left frontotemporal region (FT7); electrode placement involved a central anode electrode over FT7 and four surrounding cathode electrodes F7, T7, FC5, FT9 (−0.375 mA each) arranged in a ring configuration. Figure 3 displays the predicted current flow and electrode montage. Sham involved a 30 s ramp-up/ramp-down stimulation procedure using the same montage as active (Palm et al., 2013) which enabled blinding of the stimulation condition.

### Constraint Induced Language Therapy (CILT)

Constraint induced language therapy is a behavioral SLT that targets speech production by requiring communicative interactions using only spoken output while constraining the use of non-verbal forms of communication, such as gesturing (Maher et al., 2006). Participants used spoken output to request cards depicting everyday objects and actions (Kirmess and Maher, 2010; Ciccone et al., 2015). If unsuccessful, a semantic cue was provided, followed by a phonetic cue, then the correct response. Over the course of therapy, difficulty increased from single noun production (object stimuli) to a single verb response (action stimuli) followed by generating sentences for the object and action stimuli. CILT administration followed prior studies, as described elsewhere (Pulvermüller et al., 2001; Maher et al., 2006).

### Western Aphasia Battery-Revised (WAB-R) Parameters

The WAB-R is a widely employed, standard assessment of aphasia characteristics and severity that shows high test-retest reliability (Kertesz, 2006). The WAB Aphasia Quotient (WAB-AQ)–a measure of overall severity–served as a primary outcome measure. WAB subtests were examined separately for spontaneous speech, auditory-verbal comprehension, repetition, and naming. Imaging analyses focused on outcome measures demonstrating stimulation-induced changes from baseline.

### Statistical Analyses

#### Western Aphasia Battery-Revised Behavioral Performance

Repeated-measures analyses of variance (RM-ANOVAs) were conducted on change scores reflecting the difference between baseline to 0- and 6-week post-treatment to eliminate confounds from potential carry over effects on the WAB-AQ and WAB subtests, with tDCS condition (active, sham) and time point (0-week, 6-week) as within-subject factors. An additional analysis was performed with randomization order (first arm: active, sham) as a between-subject factor. Data were analyzed with SPSS v26.

#### Identifying Cortical Predictors of tDCS Effects

Using a two-step approach, we conducted multiple linear regression analyses to explore (1) the relationship between tDCS-induced language gains and baseline cortical thickness or volume as predictors of treatment response for outcome measures demonstrating significant improvement; and (2) the predictive value of significant ROIs at each time point while accounting for global left hemisphere and whole brain thickness and volume.

Given the exploratory nature of these analyses, we conducted separate backward-stepwise regression analyses including all seven ROIs for each cortical measure (thickness and volume), time point (change from 0- to 6-week), and stimulation condition (active and sham). The backward regression enabled the assessment of all variables in a single model and removed each ROI from subsequent models to identify the best model fit.

To ensure that the findings, were not driven by overall brain atrophy, additional regression analyses utilizing the enter approach were conducted on significant regions alongside covariates in the models (global left hemisphere and whole brain cortical thickness and volume).

Lastly, similar regression analyses were performed on a control region, (i.e., occipital cortex; 5 ROIs total for thickness and volume). Additional control analyses were performed using Bayesian linear regression in JASP v0.16.

## Results

### Behavior

No significant change in WAB-AQ as a function of tDCS condition was observed [*F*(1,10) = 2.53, *p* = 0.14], although there was a numerical increase from mean baseline WAB-AQ to 0-week post-intervention following active [M(±SD) = 83.20(±9.57) to 85.49(±8.63)] compared to sham stimulation [M(±SD) = 82.28(9.75) to 83.53(10.19)]. The WAB-R naming subtest revealed a significant effect of tDCS condition [*F*(1,10) = 7.11, *p* = 0.02, η_p_^2^ = 0.42; power = 0.68], reflecting performance gains in the active condition at the 0-week [M(±SD) = 0.6(0.42)] and 6-week [M(±SD) = 0.21(0.63)] time points when compared to sham [0-week: M(±SD) = 0.21(0.13); 6-week: M(±SD) = 0.03(0.06); see Table 2 for WAB scores and Supplementary Table 1 for WAB statistics]. By contrast, change in performance on the auditory-verbal comprehension subtest was significantly lower in the active versus sham condition [*F*(1,10) = 6.42, *p* = 0.03]. However, further assessment suggests this finding does not represent meaningful change. Specifically, baseline scores in the active condition tended to be closer to ceiling. Furthermore, including randomization order as a covariate yielded no effect of stimulation condition (*p* = 0.06). No other WAB-R subtests significantly changed (*p*’s > 0.05). This and the finding that the auditory-verbal comprehension subset slightly, but significantly, declined following active versus sham stimulation potentially explains the lack of tDCS-induced changes on overall WAB-AQ. Randomization order was not significant in any analysis (*p*’s > 0.05). Lastly, there were no significant differences between baseline arm 1 versus arm 2 (paired samples *t*-test *p* > 0.8).

**TABLE 2:** Summary of means and standard deviations (SD) on the WAB-AQ and WAB subtests.

WAB-AQ and subtests	Active			Sham		
	Baseline Mean (SD)	0-week Mean (SD)	6-week Mean (SD)	Baseline Mean (SD)	0-week Mean (SD)	6-week Mean (SD)
Aphasia quotient (AQ)	83.20 (9.57)	85.49 (8.63)	82.74 (9.92)	82.28 (9.74)	83.53 (10.18)	82.98 (10.01)
Spontaneous speech	16.94 (2.19)	17.50 (1.78)	16.91 (1.97)	16.72 (2.08)	16.92 (2.07)	16.75 (2.05)
Auditory verbal comprehension	9.38 (0.67)	9.32 (0.64)	9.29 (0.90)	9.18 (0.68)	9.38 (0.52)	9.38 (0.56)
Repetition	7.68 (1.46)	7.78 (1.68)	7.63 (1.71)	7.65 (1.50)	7.68 (1.50)	7.75 (1.70)
Naming	7.54 (2.36)	8.14 (2.22)	7.55 (2.30)	7.59 (2.25)	7.80 (2.38)	7.62 (2.31)

### Cortical Thickness and Volume

#### Backward-Stepwise Linear Regressions

**FIGURE 4:**
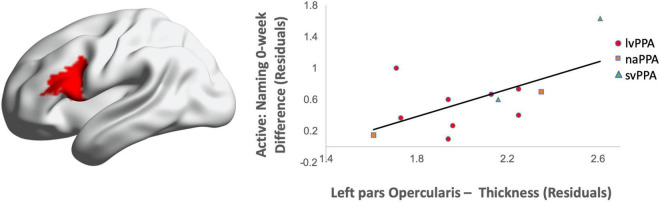
Greater baseline cortical thickness of the IFGop (highlighted in red) significantly predicted naming gains in the active condition at 0-week post-intervention (red = greater thickness; *R*^2^ = 0.32).

**FIGURE 5:**
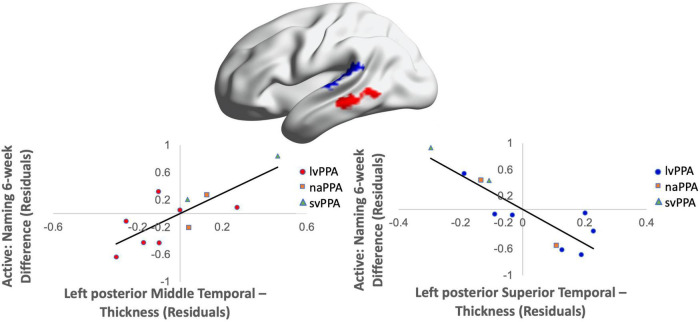
Greater baseline cortical thickness of the MTG (highlighted in red) and lower thickness of the pSTG (highlighted in blue) predicted naming gains in the active condition at 6-week post-intervention (red = greater thickness; blue = lower thickness; *R*^2^ = 0.72).

Greater cortical thickness of the IFGop significantly predicted naming gains at the 0-week time point in the active condition [*F*(1,10) = 6.08; *b* = 0.62; *p* = 0.03; *R*^2^_Adjusted_ = 0.32; Figure 4]. Analyses of naming gains 6-week post-active stimulation revealed two significant predictors of tDCS-induced naming gains: greater cortical thickness of the pMTG and lower thickness of the pSTG [*F*(4,6) = 7.46; *b* = 0.92, −0.202; *p* = 0.02; *R*^2^_Adjusted_ = 0.72; Figure 5]. Cortical thickness did not significantly predict naming performance following sham stimulation at either time point (*p*’s > 0.05).

**FIGURE 6:**
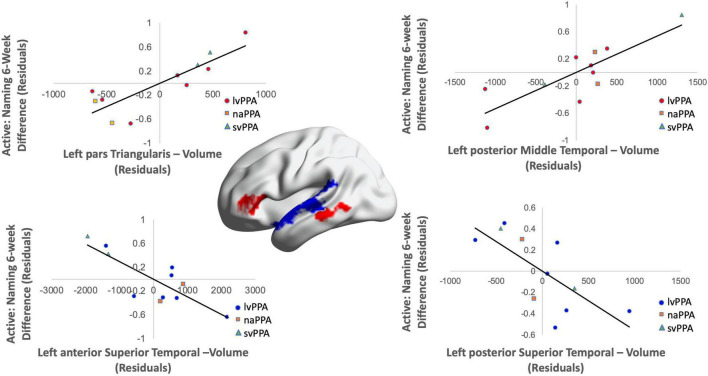
Greater baseline cortical volume of the IFGtr (highlighted in red), MTG (highlighted in red), and lower volume of aSTG and pSTG (highlighted in blue) predicted naming gains in the active condition at 6-week post-intervention (red = greater thickness; blue = lower thickness; *R*^2^ = 0.74).

**FIGURE 7:**
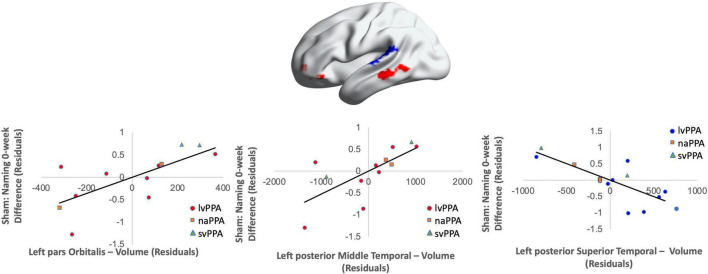
Greater baseline cortical thickness of the IFGorb (highlighted in red), MTG (highlighted in red), and lower thickness of the pSTG (highlighted in blue) predicted sham naming gains at 0-week post-intervention (red = greater thickness; blue = lower thickness; *R*^2^ = 0.56).

Cortical volume did not predict naming performance 0-week post-active stimulation (*p*’s > 0.05); however, baseline cortical volume of frontal and temporal regions significantly predicted naming gains at 6-week post-intervention: greater left IFGtr and pMTG volume and lower aSTG and pSTG volume [*F*(4,6) = 8.01; *b* = 0.76, 0.71, −0.57, −0.70; *p* = 0.014; *R*^2^_Adjusted_ = 0.74; Figure 6]. In the sham condition, at the 0-week time point, greater cortical volume of the IFGorb and pMTG, but lower volume of the pSTG predicted naming gains [*F*(3,8) = 5.71; *b*’s = 0.697, 0.613, −0.836; *p* = 0.02; *R*^2^_Adjusted_ = 0.56; Figure 7]. Cortical volume did not significantly predict naming performance 6-week post-sham (*p*’s > 0.05).

See Table 3 for a complete summary of regression model results. See Supplementary Figures 1, 2 for nonsignificant sham results.

**TABLE 3:** Summary of linear regression (backward-fitted model) results for significant thickness and volume ROIs at the 0-week and 6-week time points.

Condition	Measure	Time	ROI	*F*	Beta	*p*-value	*R*^2^ adjusted	Variance explained?
	Thickness	0-week	Pars opercularis	(1, 10) = 6.08	0.62	0.03*	0.32	37.80%
	Thickness	6-week	pMTG; pSTG	(4, 6) = 7.46	0.92; −2.02	0.02*	0.72	83.30%
ACTIVE	Volume	0-week	Pars orbitalis; pMTG, pSTG	(7, 4) = 0.617	0.295; 0.243; −0.09	0.73	−0.32	51.90%
	Volume	6-week	Pars triangularis; pMTG; pSTG; aSTG	(4, 6) = 8.01	0.76; 0.71; −0.57; −0.70	0.014*	0.74	84.20%
	Thickness	0-week	Pars opercularis	(1, 10) = 4.62	0.56	0.06	0.25	31.50%
	Thickness	6-week	pMTG, pSTG	(7, 4) = 0.755	−0.35; −0.58	0.65	−0.19	56.90%
SHAM	Volume	0-week	Pars orbitalis; pMTG, pSTG	(3, 8) = 5.71	0.697; 0.613; −0.836	0.02*	0.56	68.20%
	Volume	6-week	Pars triangularis; pMTG; pSTG; aSTG	(7, 4) = 1.05	0.105; 0.311; −0.90; −0.23	0.51	0.31	64.80%

Bolded values indicate significant results. *indicates p-value < 0.05.

#### Linear Regressions With Covariates

Global (total left hemisphere and whole brain) cortical thickness measures did not improve model fit above and beyond the significant ROIs that predicted active 0-week naming gains (IFGop; *p* > 0.05) or active 6-week naming gains (IFGtr, IFGorb, pMTG, pSTG; *p*’s > 0.05).

Similarly, global brain volume measures did not improve model fit above and beyond the significant ROIs that predicted active 6-week naming gains (IFGtr, pMTG, aSTG, pSTG; *p* > 0.05). Thus, disease severity expressed in volume loss does not explain tDCS-induced naming gains observed 6-week post-stimulation. Lastly, adding the global covariates to the model containing significant ROIs for the 0-week sham condition (IFGorb, pMTG, pSTG) did not improve model fit (*p* > 0.05). Together, these results provide further support for cortical thickness and volume of specific ROIs mediating short- and long-term active tDCS-induced naming gains.

#### Control Regions of Interest

Cortical thickness and volume of occipital cortex assessed as control ROIs did not significantly predict language gains at either the 0- or 6-week time points in either stimulation condition (*p*’s > 0.05). Similarly, Bayesian linear regression results confirm anecdotal to moderate evidence indicating an absence of effect in occipital ROIs (thickness and volume for active: 0-week BF_10_ = 0.36 and BF_10_ = 0.75, 6-week BF_10_ = 0.17 and BF_10_ = 0.20, respectively; sham 0-week BF_10_ = 0.19 and BF_10_ = 0.29, 6-week BF_10_ = 0.27 and 6-week BF_10_ = 0.23, respectively).

## Discussion

The current study examined whether cortical thickness or volume of left hemisphere brain structures involved in language processing can predict tDCS-induced naming improvements in persons with PPA when compared to a control region and global atrophy measures more generally. Consistent with previous literature (Tsapkini et al., 2014, 2018; Cotelli et al., 2016; McConathey et al., 2017; Ficek et al., 2018; Fenner et al., 2019), we identified a significant enhancement in naming performance after active stimulation that was not observed in the sham condition. Baseline cortical thickness and volume of frontal and temporal regions involved in language predicted tDCS-induced enhancements in naming. Differential effects were observed at post-intervention time points such that improved naming performance was associated with greater cortical thickness and volume in certain regions (IFGop, IFGtr, MTG) and lower cortical thickness and volume in other regions (aSTG, pSTG). Moreover, baseline cortical thickness predicted naming gains immediately after therapy in anterior frontal regions and included posterior temporal regions at 6-week post-intervention. This suggests that a broader network may be necessary in the maintenance of tDCS-induced treatment gains. Collectively, these findings indicate that baseline cortical thickness and volume in language processing centers may be important factors for predicting response to brain stimulation in persons with PPA.

Cortical thickness is thought be a proxy for the integrity of the cerebral cortex and relates to the size, number, and density of cells in cortical structures (Rakic, 1988; Dickerson and Wolk, 2012), while volume is thought to be a proxy of neural reserve (Arenaza-Urquijo et al., 2013), or the extent to which the brain can harbor neuropathology without an individual experiencing functional impairments (Stern, 2002, 2010; Murray et al., 2011; Medaglia et al., 2017). Correspondingly, neuroimaging studies in patients with neurodegenerative disorders demonstrate that cortical thickness associates with neuronal loss (Shefer, 1973; Fischl and Dale, 2000), while lower volume associates with worse cognitive performance (Vollmer et al., 2016). Thus, our primary focus was on cortical thickness and volume of the left hemisphere language network to demonstrate whether baseline cortical measures could predict treatment-induced language gains. The fact that we identified strong correlations between cortical thickness and volume in language-relevant brain regions and tDCS-induced naming benefits experienced by persons with PPA aligns well with the roles that these brain areas are understood to have in naming and in language processing more broadly.

The IFG, a critical region for speech production, includes the two components of Broca’s area, the IFGop and IFGtr (Foundas et al., 1998; Holland et al., 2011). IFG recruitment occurs during propositional speech, verb generation (Blacker et al., 2006; Costafreda et al., 2006; Harrington et al., 2006), picture naming (Moore and Price, 1999; Harrington et al., 2006; Breier and Papanicolaou, 2008; Delikishkina et al., 2020), and action observation (Molnar-Szakacs et al., 1991). Given the essential nature and abundant connections of the IFG to other language-relevant regions (Friederici and Gierhan, 2013; Hertrich et al., 2020), it may be an important target for stimulation. Here, greater baseline cortical thickness and volume of the IFGop and IFGtr, respectively, associated with naming improvement after active, but not sham, stimulation. These findings suggest that spared or partially spared regions within the IFG are necessary for tDCS-induced enhancement of naming performance. Moreover, these findings lend support for targeting the IFG in the context of brain stimulation, and similarly are consistent with previous research showing that active tDCS over the IFG paired with SLT conferred improvements in naming and other aspects of language in PPA patients (Webster et al., 2016; Fenner et al., 2019; de Aguiar et al., 2020).

We also observed predicted results in the MTG, but encountered surprising results in the STG. Specifically, greater MTG but lower STG thickness and volume associated with naming gains in the active group. Neuroimaging studies demonstrate MTG activation during lexico-semantic retrieval (Baldo and Dronkers, 2006; Oppenheim et al., 2010; Harvey and Schnur, 2015), picture naming (Indefrey and Levelt, 2004), and processing complex action knowledge (Wallentin et al., 2011). This suggests that increased thickness and volume of the MTG may allow for stimulation-induced enhancements of semantic-to-lexical mapping critical for picture naming tasks (Schwartz et al., 2009). The STG, which is involved in word recognition and naming, comprises Wernicke’s area, a region vital for language comprehension. The findings that lower cortical measures of the STG predicted naming improvements in active group was somewhat unexpected given that regions with greater cortical atrophy have shown associations with decrements in language abilities (Rogalski and Mesulam, 2009; Rogalski et al., 2011; Rohrer, 2012; Rohrer et al., 2013). One explanation for the negative correlation between naming gains and lower STG thickness and volume may be that there is greater capacity for tDCS-induced improvement in individuals with greater baseline impairment. This aligns with previous research demonstrating that more impaired PPA patients at baseline may respond more positively to tDCS-related language therapies (McConathey et al., 2017). Our results also align with research demonstrating that lower baseline cortical volume of some language-related regions predicted improvement in written naming and spelling after tDCS intervention targeting the IFG in PPA patients (de Aguiar et al., 2020). In that study, letter accuracy improvements observed in active, but not sham, tDCS administered during therapy were associated with lower volume of the left angular gyrus (de Aguiar et al., 2020). Prior research in healthy participants has also confirmed that individual differences in cortical morphology of the left versus right prefrontal cortex associate with differences of tDCS effects on cognition in the context of decision-making (Filmer et al., 2019).

This study has several limitations. The sample size was small (*N* = 12) which reduces statistical power. However, this sample size is in line with previously published research involving tDCS in PPA patients that also report positive results on language tasks (Gervits et al., 2017; Fenner et al., 2019; Hosseini et al., 2019), suggesting it is a promising intervention in this population. It is possible that stimulation could increase general arousal. We find this unlikely in the current study, as previous research in healthy individuals assessing tDCS on semantic and phonemic fluency after left IFG stimulation have shown that active versus sham stimulation increases word production and control experiments confirmed that these findings were not dependent on levels of general arousal (Cattaneo et al., 2011). Although all PPA variants were included, the majority of participants were of the lvPPA subtype. Thus, differences in neuroanatomical predictors of stimulation effects across variants could not be assessed; results may not be as applicable to naPPA and svPPA patients. The range in values for lvPPA patients suggest results are not driven by categorical differences between variant subtype, but rather reflect variability predominantly in the lvPPA patients. Randomization was not well balanced for stimulation condition within and across variants. The current study findings are preliminary and should be validated in a larger sample of PPA patients with equal variant subtypes, severity, and randomization of stimulation condition. We selected cortical areas for analysis that are central in language processing, but it is possible that different regions in the language network would better predict tDCS-induced naming improvement. However, this explanation is unlikely because the inclusion of all left hemispheric cortical regions and the whole brain as covariates in our models as well as analysis of a control region, i.e., the occipital lobe, did not yield significant results, suggesting that other candidate left-hemispheric language regions or disease severity more generally do not account for the current findings. Thus, the functional role of stimulated regions and their connectivity with other regions likely plays an important role in determining response to stimulation.

## Conclusion

Differential findings were observed in terms of thickness and volume, where some regions with greater or lower cortical measures at baseline were predictive of naming improvements after intervention. Nevertheless, these findings indicate that the degree and location of atrophy are important factors in response to naming-based enhancement from tDCS in PPA. Our findings provide insight into cortical predictors of tDCS-induced naming gains and lend support for stimulation of the left IFG as a promising target for improving SLT outcomes in persons with PPA. Taken together, these results provide guidance toward the application of HD-tDCS in PPA for rehabilitation protocols.

## Data Availability Statement

The original contributions presented in this study are included in the article/Supplementary Material, further inquiries can be directed to the corresponding author.

## Ethics Statement

The studies involving human participants were reviewed and approved by the University of Pennsylvania Institutional Review Board (IRB). The patients/participants provided their written informed consent to participate in this study.

## Author Contributions

NN was responsible for the data analyses, figures, and prepared the original draft of the manuscript. DH aided in data analyses, figures, and manuscript edits. CH and LF aided in data acquisition and contributed to the original draft of the manuscript. PB, GL, JP, KC, and MG contributed to the neuroimaging acquisition, preprocessing, and edits the manuscript. SX aided in data analyses. RH was responsible for the intellectual contribution to the study design and data acquisition, and contributed to the data analyses, figures, and manuscript edits. All authors reviewed and approved the final manuscript.

## Conflict of Interest

The authors declare that the research was conducted in the absence of any commercial or financial relationships that could be construed as a potential conflict of interest.

## Publisher’s Note

All claims expressed in this article are solely those of the authors and do not necessarily represent those of their affiliated organizations, or those of the publisher, the editors and the reviewers. Any product that may be evaluated in this article, or claim that may be made by its manufacturer, is not guaranteed or endorsed by the publisher.
