# Supplementary material for: Through Thick and Thin: Baseline Cortical Volume and Thickness Predict Performance and Response to Transcranial Direct Current Stimulation in Primary Progressive Aphasia
Source: Front Hum Neurosci. 2022 Jul 7;16:907425. doi: 10.3389/fnhum.2022.907425 (PMC9302040; doi:10.3389/fnhum.2022.907425)
Supplement: Supplementary file 1 [file Table_1.DOCX]

| **Behavioral Analyses Results for the WAB and WAB Subtests** | | | | |
| --- | --- | --- | --- | --- |
|  |  |  |  |  |
|  | ***(df) F*** | ***p*** | **Partial Eta-squared** |  |
| WAB AQ |  |  |  |  |
| Stimulation Condition | (1,10) 2.525 | 0.14 | 0.202 |  |
| Time Point | (1,10) 6.818 | 0.03 | 0.405 |  |
| Stimulation Condition x Time Point | (1,10) 0.678 | 0.43 | 0.063 |  |
| WAB Spontaneous Speech |  |  |  |  |
| Stimulation Condition | (1,10) 0.686 | 0.43 | 0.064 |  |
| Time Point | (1,10) 5.213 | 0.05 | 0.343 |  |
| Stimulation Condition x Time Point | (1,10) 1.000 | 0.34 | 0.091 |  |
| WAB Auditory Verbal Comprehension |  |  |  |  |
| Stimulation Condition | (1,10) 6.421 | 0.03 | 0.391 |  |
| Time Point | (1,10) 0.007 | 0.93 | 0.001 |  |
| Stimulation Condition x Time Point | (1,10) 0.029 | 0.87 | 0.003 |  |
| WAB Repetition |  |  |  |  |
| Stimulation Condition | (1,10) 0.291 | 0.60 | 0.028 |  |
| Time Point | (1,10) 0.146 | 0.71 | 0.014 |  |
| Stimulation Condition x Time Point | (1,10) 0.052 | 0.83 | 0.005 |  |
| WAB Naming |  |  |  |  |
| Stimulation Condition | (1,10) 7.111 | 0.02 | 0.416 |  |
| Time Point | (1,10) 8.971 | 0.01 | 0.473 |  |
| Stimulation Condition x Time Point | (1,10) 0.681 | 0.43 | 0.064 |  |
| *Note.* Statistical comparison between active vs. sham behavioral data were conducted on the mean difference score from baseline for each stimulation condition at each time point (i.e., difference from baseline to 0-week follow-up and difference from baseline and 6-week follow-up). | | | | |

**Supplementary Table 1.** Summary of WAB-AQ and WAB Subtest statistics.
