# Supplementary material for: Through Thick and Thin: Baseline Cortical Volume and Thickness Predict Performance and Response to Transcranial Direct Current Stimulation in Primary Progressive Aphasia
Source: Front Hum Neurosci. 2022 Jul 7;16:907425. doi: 10.3389/fnhum.2022.907425 (PMC9302040; doi:10.3389/fnhum.2022.907425)
Supplement: Supplementary file 2 [file Image_1.pdf]

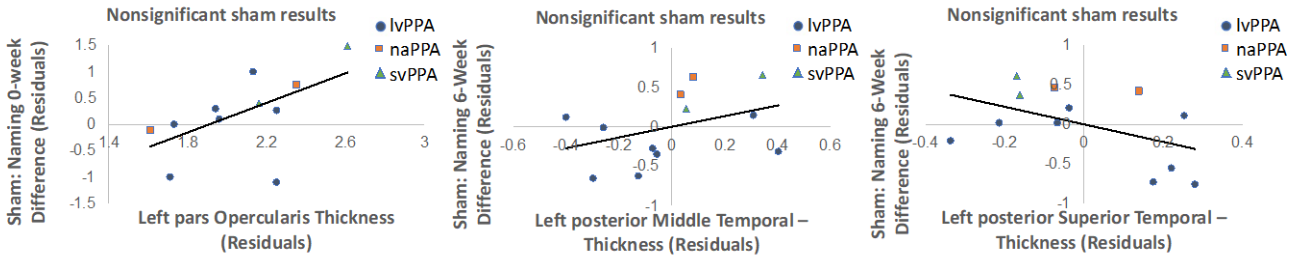

Supplementary Figure 1. Graphical summary of nonsignificant sham residuals for cortical thickness.
